# microRNA-26a represses pancreatic cancer cell malignant behaviors by targeting E2F7

**DOI:** 10.1007/s12672-021-00448-z

**Published:** 2021-11-27

**Authors:** Liang Wang, Meijun Li, Fei Chen

**Affiliations:** 1 Department of Hepatobiliary Surgery, The First Affiliated Hospital of Jinzhou Medical University, Jinzhou, China; 2 Department of Blood, The Third Affiliated Hospital of Jinzhou Medical University, Jinzhou, China; 3 Department of Ultrasound, The First Affiliated Hospital of Jinzhou Medical University, No. 2 of the People Street, Gu Ta district, Jinzhou, 121001 Liao Ning China

**Keywords:** miR-26a, Pancreatic cancer, E2F7, VEGFA, Cell proliferation

## Abstract

Dysregulation of microRNAs (miRNAs) exerts key roles in the development of pancreatic cancer (PCa). miR-26a is reportedly a tumor suppressor in cancers. However, whether miR-26a modulates PCa progression is poorly understood. Here, we found that miR-26a was down-regulated in PCa. Overexpressed miR-26a suppressed PCa cell proliferation, colony formation, and tumor stem cell properties. Mechanically, the transcription factor E2F7 is a downstream target of miR-26a. miR-26a decreased E2F7 expression through binding to the 3’-untranslated region (UTR) of E2F7. Decreased miR-26a in PCa tissues was inversely correlated with E2F7. The inhibitory effects of miR-26a in PCa were reversed by E2F7 overexpression. Consistently, the knockout of E2F7 further significantly inhibited the growth of PCa cells combined with miR-26a overexpression. Further study revealed that E2F7 bound the promoter of vascular endothelial growth factor A (VEGFA), a key factor in angiogenesis, and transcriptionally activated the expression of VEGFA. miR-26a overexpression attenuated the effects of E2F7 on VEGFA promotion. Our results uncovered the novel function of miR-26a/E2F7/VEGFA in PCa, making miR-26a a possible target for PCa treatment.

## Introduction

Pancreatic cancer (PCa) is a well-known fatal aggressive tumor with a poor prognosis [1–3]. It is the seventh leading cause of cancer-related death worldwide. Currently, surgery remains the only potentially curative treatment for patients with early pancreatic cancer [4–6]. However, for patients diagnosed at an advanced stage, the prognosis remains unsatisfactory due to a lack of efficient treatment strategy and non-specific symptoms for early diagnosis. Therefore, the identification of novel targets that accurately detect PCa and the development of novel treatment options is desirable.

MicroRNAs (miRNAs) are endogenous, single-stranded small RNA molecules that cannot be translated into proteins. miRNAs suppress the translation or promote the degradation of target mRNAs in a sequence-specific manner [7–9]. MiRNAs reportedly modulate cell growth, differentiation, and migration [10, 11]. Current research indicates up- or down-regulation of miRNAs in cancer [12–17]. In pancreatic cancer, miRNAs affect the development of PCa by targeting key factors associated with cancer progression [18–22]. Deregulated miRNAs show correlation with the diagnosis, prognosis and response to current therapies of pancreatic cancer patients [23]. Identification of novel miRNAs that are aberrantly expressed between normal and cancerous specimens have been made via microarrays or RNA-sequencing analysis [24]. More interestingly, considering the stability of miRNAs in serum, non-invasive blood screening test of the miRNAs might be a promising approach to predict the disease aggressiveness [23, 24].

Recent studies have shown that miR-137 targets KLF12 and suppresses the stemness features of pancreatic cancer cells [25]. The anti-cancer function of miR-26a is supported by a growing body of evidence [26–32]. Reduced miR-26a expression in cancer was correlated with a more aggressive patient status. Overexpressed miR-26a repressed cell proliferation and sensitized cancer cells to anti-cancer drugs. miR-26a delivery is a possible strategy to treat PCa by restoring wild-type functions to mutant p53 [33]. Besides, miR-26a was reported as a suppressor of pancreatic cancer via down-regulating cyclin E2 [34]. Lower miR-26a expression was correlated with the significantly shorter survival of pancreatic cancer patients [34]. This evidence suggests the importance and targets of miR-26a in pancreatic cancer need more investigation.

This study aimed to clarify the regulatory role of miR-26a in PCa and evaluate the possible mechanism involved. Our findings demonstrate the significantly reduced expression of miR-26a in PCa. miR-26a overexpression repressed proliferation and colony formation and induced apoptosis of PCa cells. Furthermore, a mechanism study suggested that the tumor-suppressive function of miR-26a in PCa was achieved through targeting E2F7. These findings uncovered the novel involvement of miR-26a/E2F7 axis in PCa, which suggests miR-26a is a promising anti-cancer target for PCa.

## Materials and methods

### Patient tissues and cell lines

We enrolled 50 PCa patients in this study. They provided PCa tissues and paired adjacent normal tissues via surgery at the First Affiliated Hospital of Jinzhou Medical University from January of 2012 to December of 2015. Tissues were stored in liquid nitrogen before use. The Ethics Committee of The First Affiliated Hospital of Jinzhou Medical University approved this study. Written informed consent was provided by all patients and their relatives.

We purchased AsPC-1, BXPC-3, Sw1990, and PANC-1 cell lines from the American Type Culture Collection (ATCC, Manassas, VA, USA). These cells were cultured with DMEM containing 10% fetal bovine serum (FBS, Gibco, NY, USA) with the addition of 100 U/mL penicillin and 100 μg/mL streptomycin (Gibco, NY, USA). Cells were maintained at 37 ℃ in an atmosphere with 5% CO_2_.

### RNA preparation and RT-qPCR

RNA was isolated with TRIzol reagent (Solarbio, Beijing, China) followed by reverse transcription using the MicroRNA Reverse Transcription Kit (TIANGEN, Beijing, China). RT-qPCR was carried out on the ABI 7500 Fast Real-Time PCR Platform (Applied Biosystems) with the PCR Master Mix (TIANGEN, Beijing, China). The conditions of qPCR were initiated at 94 ℃ for 5 min, followed by 39 cycles of 94 ℃ for 10 s and 58 ℃ for 30 s. U6 RNA expression was detected as the control. The primers were designed as miR-26a f, 5’-GACTGTTCAAGTAATCCAGGATA; miR-26a r, 5’-GTGCAGGGTCCGAGGTATTC; U6 RNA f, 5’-CTCGCTTCGGCAGCACA; U6 RNA r, 5’-AAACGCTTCACGAATTTG CGT. The relative expression of miR-26a was qualified with the 2^−ΔΔCT^ formula.

### Cell counting kit-8 (CCK-8) assay

CCK-8 experiments were completed to monitor PCa cell proliferation. Transfected PCa cells were seeded into the 96-well plates. The cell proliferation was measured with the CCK-8 solution (Beyotime, Shanghai, China) at 24 h intervals. After incubating with the CCK-8 for 3 h at 37 ℃, a microplate reader (Biotex, Winooski, VT, USA) was used to detect the OD450 nm absorbance of each well.

### In vitro colony formation analysis

PCa cells expressing miR-26a mimics or miR-NC were cultured in 6-well plates (~ 500 cells per well) with DMEM supplemented with FBS. After 10 days, cells were stained with 0.1% crystal violet solution (Solarbio, Beijing, China) after fixing with 100% methanol at room temperature (RT) for 15 min. Colonies were washed with PBS and counted using light microscopy.

### Western blot

An equal amount of protein was loaded and separated by running a 15% SDS-PAGE. After transfer, the membrane was blocked with 5% non-fat milk for 1 h at RT followed by incubation with a primary antibody against E2F7 (1:2000; ab56022, Abcam) or GAPDH (1:3000; ab181602, Abcam) for 2 h at RT. Fluorescently labeled secondary antibody was then applied to develop the signals using the Odyssey CLx (Li-Cor, Lincoln, NE, USA).

### Dual-luciferase activity analysis

The 3’-UTR sequence of E2F7 was constructed into the pGL3 luciferase vectors (Addgene, USA). PCa cells were co-transfected with the luciferase plasmids and miR-26a mimic or miR-NC using PEI (Solarbio, Beijing, China). To detect binding between E2F7 and VEGFA, VEGFA’s promoter sequence was inserted into the backbone of the pGL-Basic vector (Addgene, USA). After the transfection of pGL-Basic-VEGFA and pcDNA-E2F7 or the corresponding empty vector for 48 h, PCa cells were lysed to detect luciferase activity with the Dual-Luciferase Reporter Gene Assay Kit (Yeasen, Shanghai, China). The luciferase activity of the *renilla* gene was also determined for normalization. The experiment was performed in triplicate.

### Chromatin Immunoprecipitation (ChIP)-qPCR

PCa cells transfected with the indicated expression vectors were harvested after culture for 48 h. The CHIP assay was carried out as previously reported. Briefly, cross-linking was performed with 1% formaldehyde for 10 min at RT and then lyzed with protease inhibitor on ice for 10 min. Samples were then sonicated at 4 ℃ at 20 kHz to generate chromatin fragments of 200–500 bp. The DNA fragments were isolated with the MolPure Cell/Tissue DNA kit (YEASEN, Shanghai, China) and amplificated via qPCR using the TransStart Green qPCR SuperMix (Transgene, Beijing, China) with the VEGFA primers (forward, 5′-GCTGTTTGGGAGGTCAGAAATAGG and reverse, 5′-ACGCTGCTCGCTCCATTCAC). We used an antibody against E2F7 (sc-H300, Santa Curz Biotechnology, USA) and normal rabbit IgG as a negative control.

### Data analysis

Statistical analysis was performed with the SPSS 19.0 software (IBM, Armonk, NY, USA). Results were shown as mean ± standard deviation (SD). Student’s *t*-test or one-way analysis of variance (ANOVA) followed by Tukey’s post-hoc test was performed to determine significance. A *P*-value less than 0.05 was considered significant.

## Results

### MiR-26a level was decreased in PCa

**Fig. 1:**
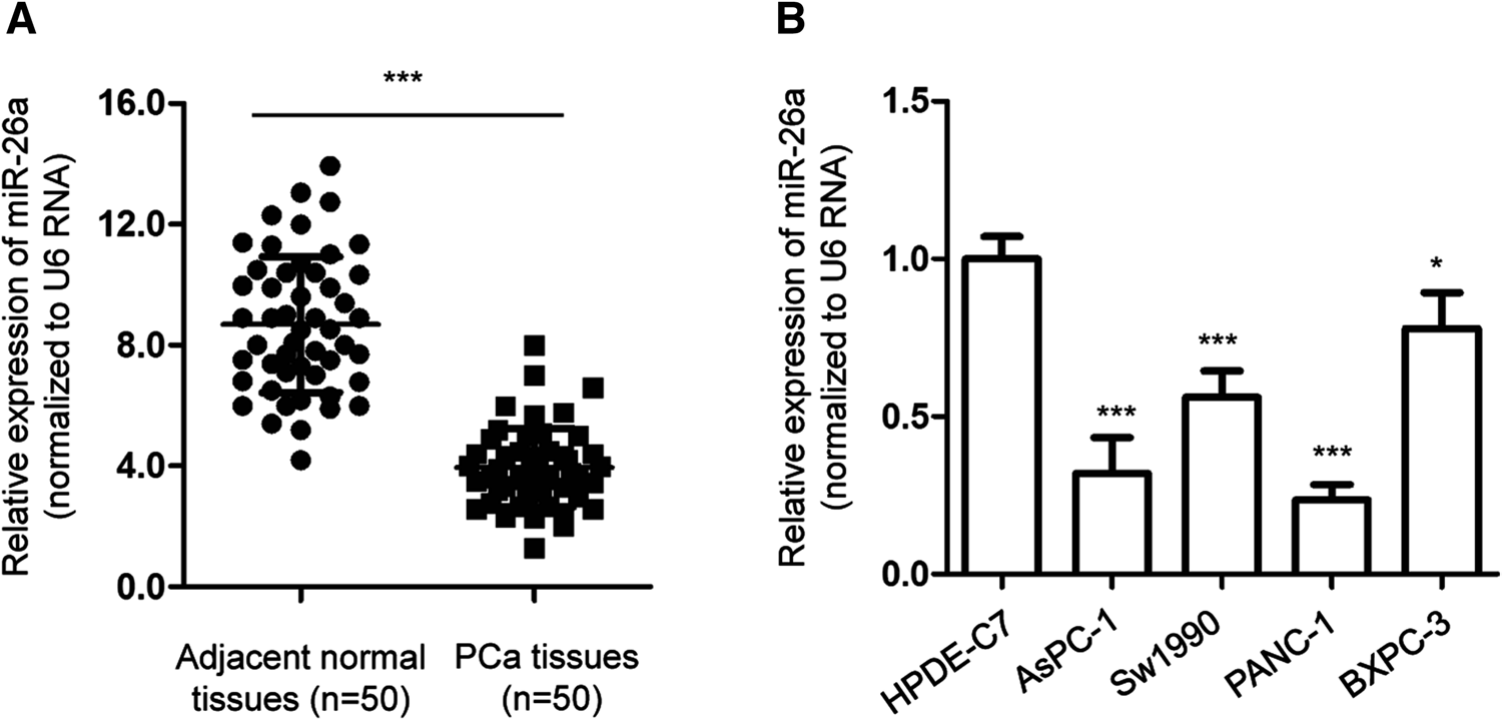
miR-26a expression was down-regulated in PCa. **A** A comparison of miR-26a expression in 50 paired PCa tissues and adjacent non-cancer tissues. **B** miR-26a levels in normal HPDE-C7 cell and PCa cell lines. Sw1990, PANC-1, AsPC-1, and BXPC-3. *P < 0.05; ***P < 0.001

To explore the potential involvement of miR-26a in PCa, the expression of miR-26a was detected by RT-qPCR in 50-paired PCa tissues and adjacent non-cancerous tissues. As presented in Fig. 1A, a significantly decreased miR-26a expression was obtained in PCa tissues. Additionally, the expression of miR-26a was also evaluated in PCa cells and the normal cell HPDE6-C7. As indicated in Fig. 1B, lower miR-26a levels were more frequently observed in PCa cells than in normal cells. This indicates that there is a reduced miR-26a abundance in PCa.

### Overexpression of miR-26a suppressed the malignant behaviors of PCa cells

**Fig. 2:**
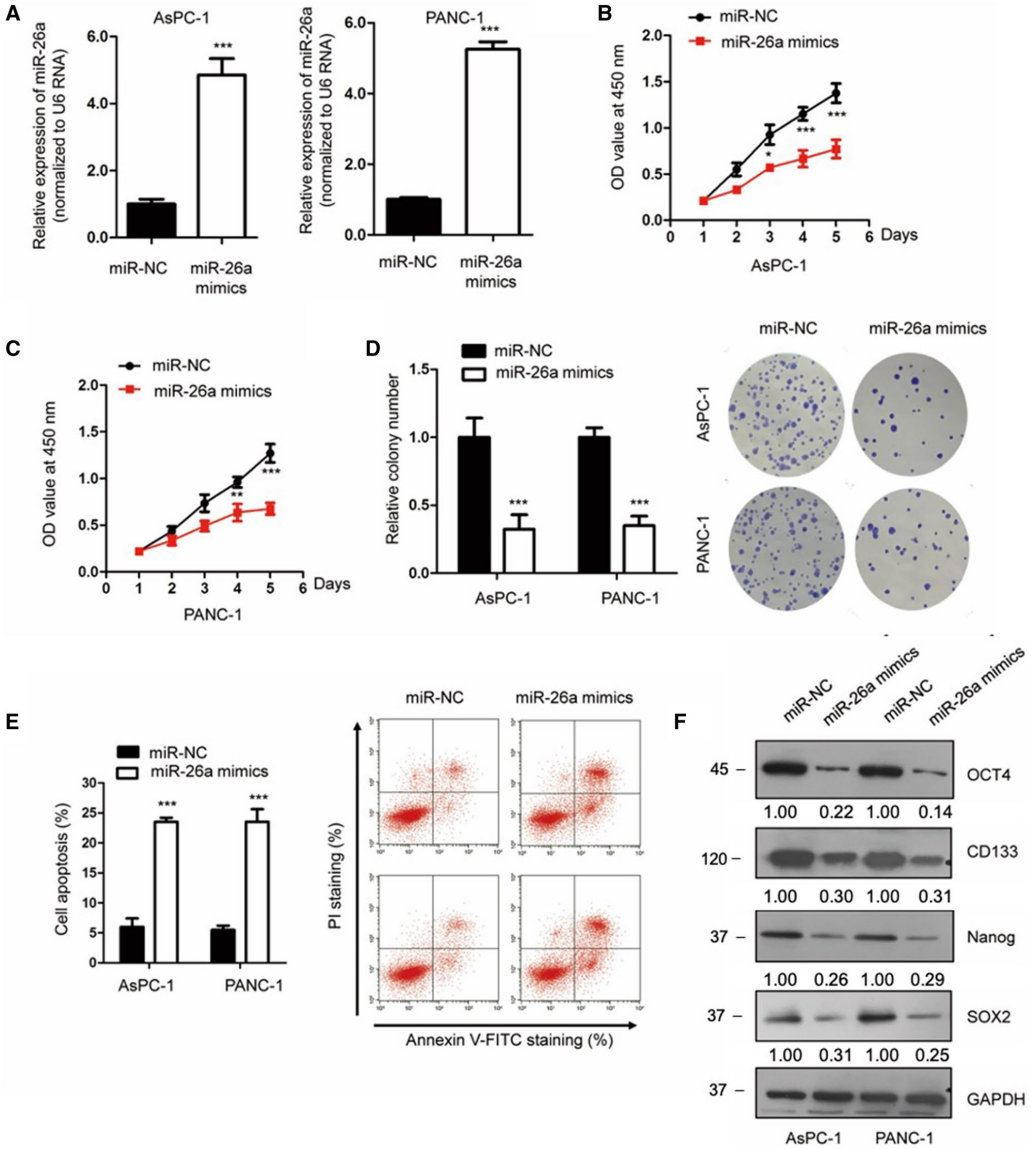
miR-26a suppressed the proliferative ability of PCa cells. **A** miR-26a expression of AsPC-1 and PANC-1 cells after miR-26a transfection. **B**, **C** The proliferation of PCa cells with overexpression of miR-26a was significantly inhibited. **D** miR-26a transfection decreased the number of PCa cells. **E** miR-26a enhanced apoptosis of both AsPC-1 and PANC-1 cells. **F** The expression levels of the stemness markers in PCa cells were examined after transfection of miR-26a. *P < 0.05; **P < 0.01; ***P < 0.001

Considering the down-regulation of miR-26a in PCa, we further investigated the biological roles of miR-26a via gain-of-function experiments. The miR-26a expression in PCa cells was significantly increased following the transfection of miR-26a mimics (Fig. 2A). The CCK-8 data in Fig. 2B and C indicate that the proliferation of PANC-1 and AsPC-1 cells was repressed with miR-26a. The time that cell number doubled the initial in control and miR-26a groups was 1.95 days and 2.80 days, respectively (Fig. 2B). For Fig. 2C, the values for control and miR-26a groups were 2.02 and 3.13 days, respectively. Transfection of miR-26a also resulted in a reduced colony number of both AsPC-1 and PANC-1 cells (Fig. 2D). Moreover, increased miR-26a expression promoted the apoptosis of PCa cells (Fig. 2E). Increasing evidence suggests that tumor stem cells play important roles in regulating tumorigenesis [35, 36].

To determine whether miR-26a affected PCa cell growth via modulation of the stemness, we examined the expression of OCT4, CD133, Nanog, and SOX2, the stem-cell-like markers. miR-26a overexpression reduced the expression of these four stem-cell markers in PCa cells when compared to the control group (Fig. 2F), which suggests inhibited stem cell properties of PCa cells with overexpressed miR-26a. miR-26a inhibits the malignant behaviors of PCa.

### miR-26 suppressed the expression of E2F7 in PCa cells

**Fig. 3:**
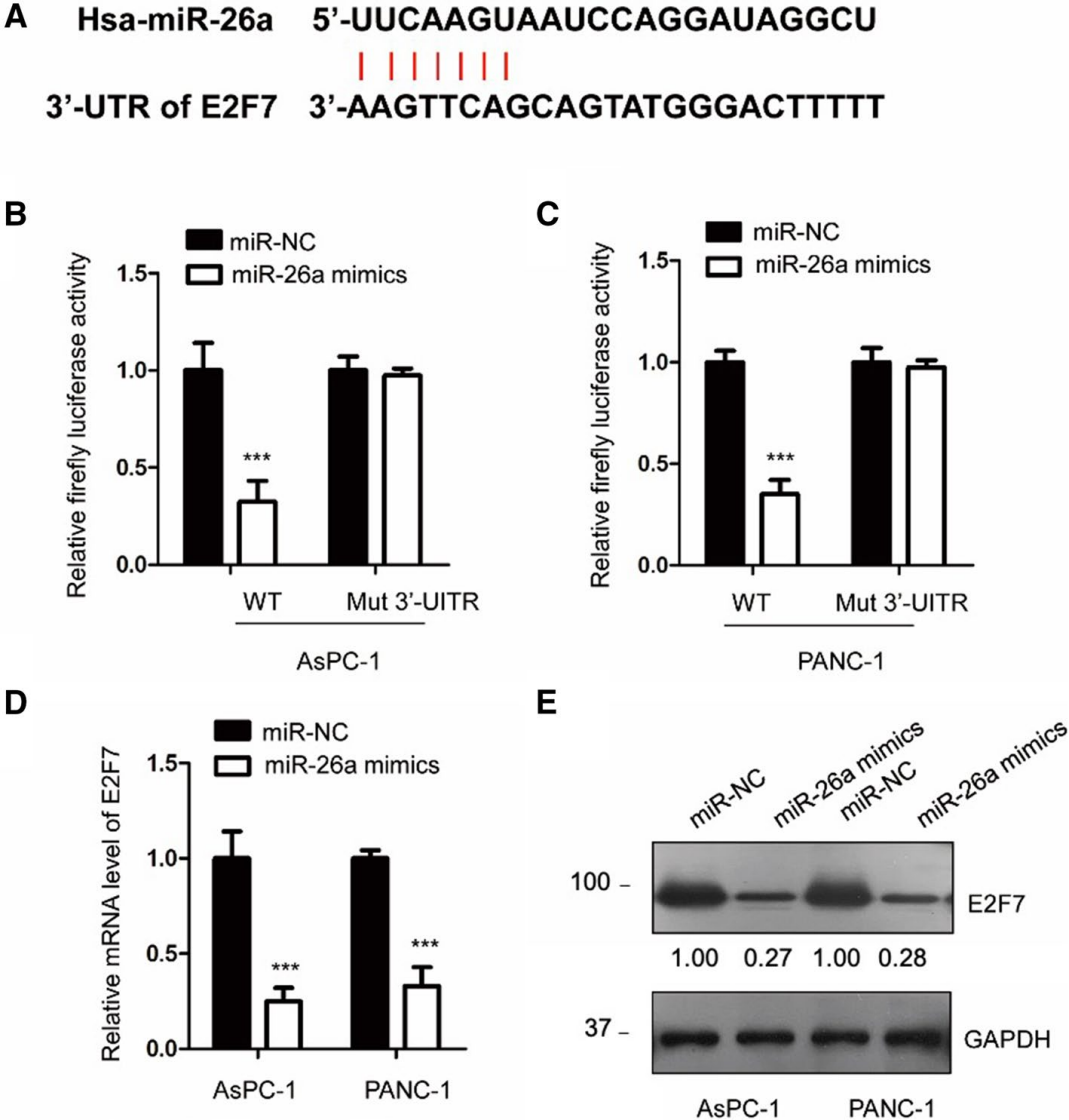
miR-26a targeted E2F7 in PCa. **A** The possible binding site of miR-26a in the 3’-UTR of E2F7 mRNA. **B**, **C** The luciferase activity of AsPC-1 and PANC-1 cells that were co-transfected with miR-26a and either vector expressing WT or Mut 3’-UTR of E2F7. **D**, **E** We detected the mRNA or protein expression of E2F7 in PCa cells that were overexpressed with miR-26a. ***P < 0.001

Previous studies reported that miR-26a regulated cancer progression by targeting the 3’-UTR of downstream mRNAs. We searched for miR-26a candidate targets using an online database and 1134 candidates were found. Among them, E2F7 ranked top 21 with the Target Score 99. As a member of E2F transcription factors, E2F7 is essential for regulating the cell cycle. Overexpression and oncogenic function of E2F7 have been well established in cancers, including lung cancer, rectal adenocarcinoma, papillary thyroid cancer, esophageal cancer, and gastric cancer [37]. The prognostic value of E2F7 in pancreatic cancer was also emerged [38], however, the role of E2F7 in pancreatic cancer still remains largely unknown. Based on these points, we chose E2F7 as a target of miR-26a in this study. A highly scored binding site of miR-26a within the 3’-UTR of E2F7 was presented in Fig. 3A. This prediction was further confirmed by the luciferase reporter assay. As indicated in Fig. 3B and C, miR-26a significantly decreased the luciferase activity via binding to the 3’-UTR of E2F7 (Fig. 3B and C).

However, a mutation in E2F7 3’-UTR that abolished its binding with miR-26a did not respond to miR-26 s. We used RT-qPCR to detect the mRNA levels of E2F7 in PCa cells that were transfected with a miR-26a mimic or corresponding scramble miRNA to determine whether this binding could affect E2F7 mRNA abundance. As indicated in Fig. 3D, the mRNA abundance of E2F7 was significantly lower with miR-26a than the control. Consistently, overexpressing miR-26a in both PANC-1 and AsPC-1 cells inhibited E2F7 protein expression (Fig. 3E). These results demonstrated that reduced E2F7 by miR-26a might be responsible for the inhibitory function of miR-26a in PCa.

### E2F7 mediated the inhibitory function of miR-26a in PCa

**Fig. 4:**
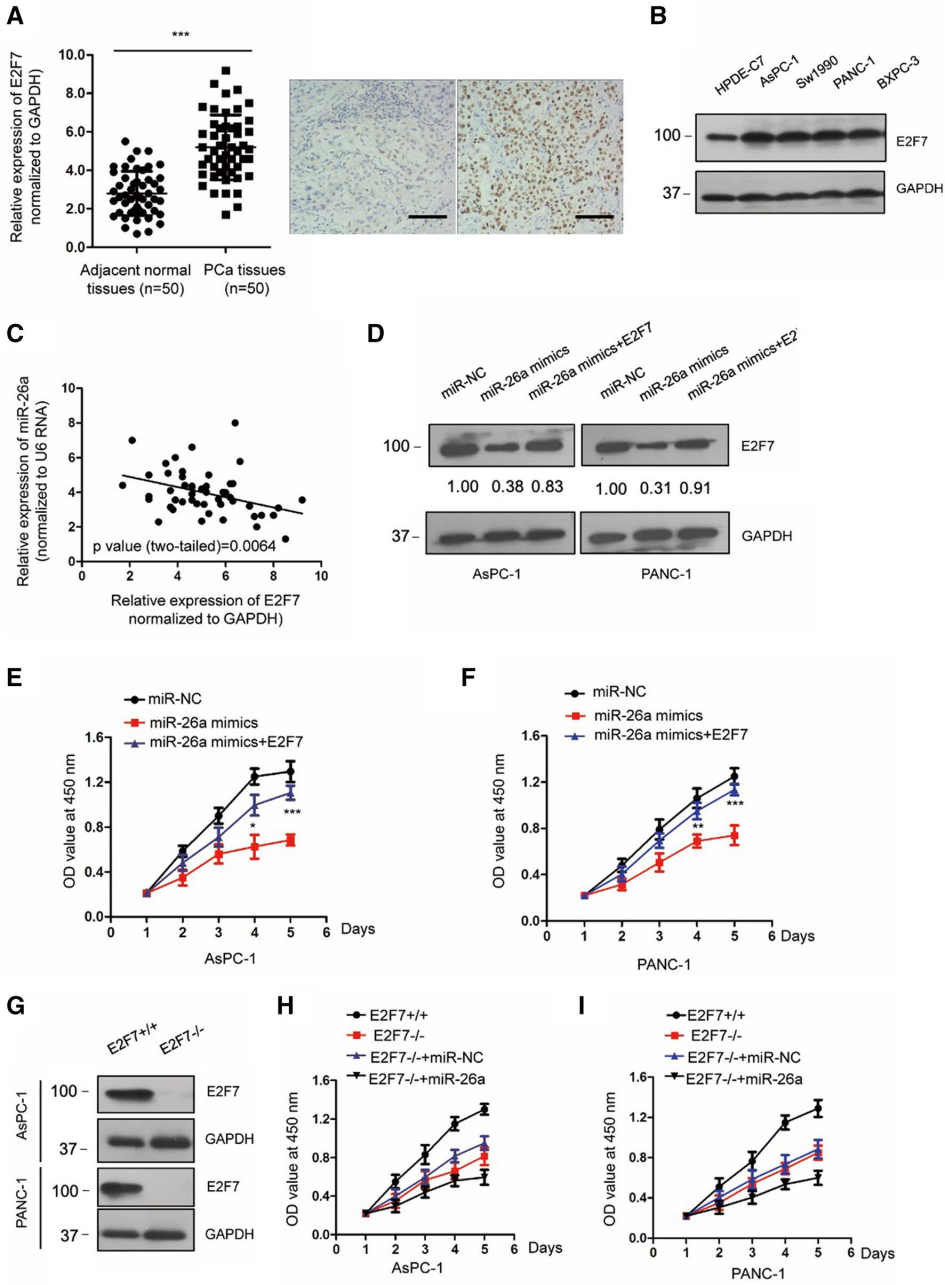
Overexpressed E2F7 in PCa attenuated the suppressive role of miR-26a. **A** E2F7 mRNA levels in the indicated tissues samples were detected using RT-qPCR. The representative IHC staining of VEGFA in PCa tissues and adjacent normal tissues. Scale bar, 50 um. **B** The protein levels of E2F7 in normal and PCa cells were compared by Western blot. **C** Spearman correlation test showed the inverse correlation between miR-26a and E2F7 in PCa. **D** We transfected PCa cells with the indicated vectors and detected E2F7 expression. **E**, **F** Reintroduction of E2F7 significantly reversed the suppressed proliferation by miR-26a. **G** The endogenous level of E2F7 was knocked out by Caspir-cas9, and the silencing efficiency was confirmed via Western blotting. **H**, **I** Silencing of E2F7 expression further inhibited PCa cell proliferation by miR-26a overexpression. *P < 0.05, **P < 0.01, and ***P < 0.001

To explore the role of E2F7 in miR-26a-mediated growth inhibition of PCa, we performed RT-qPCR to quantify E2F7 expression in the cohort of tissues that were used for detecting miR-26a. Compared with the expression level of the control group, a significantly higher level of E2F was observed in PCa tissues via both RT-qPCR and immunohistochemistry staining (Fig. 4A). Moreover, higher E2F7 abundance in PCa cells was also found (Fig. 4B). Considering the negative regulation of E2F7 by miR-26a, the correlation between the expression of miR-26a and E2F7 was evaluated by the Spearman test. Figure 4C indicates the inverse correlation between levels of E2F7 and miR-26a in PCa tissues.

To further investigate the involvement of E2F7 in PCa, the expression plasmid of E2F7 was transfected into both PANC-1 and AsPC-1 cells with the combination of miR-26a mimics (Fig. 4D). Interestingly, the suppressed proliferation of PCa cells by miR-26a was attenuated with the reintroduction of E2F7 (Fig. 4E and F). To further investigate the critical function of E2F7, E2F7 was knocked out by CRISPR and confirmed by Western blot (Fig. 4G). Consistently, combined E2F7 knockout and miR-26a overexpression further inhibited PCa cell proliferation in compassion with the parental E2F7-/- cells (Fig. 4H and I). These results suggest that reduced E2F7 was at least partially responsible for the anti-cancer effects of miR-26a in PCa.

### miR-26a transcriptionally inhibited VEGFA by suppressing the binding of E2F7 with the promoter of VEGFA

**Fig. 5:**
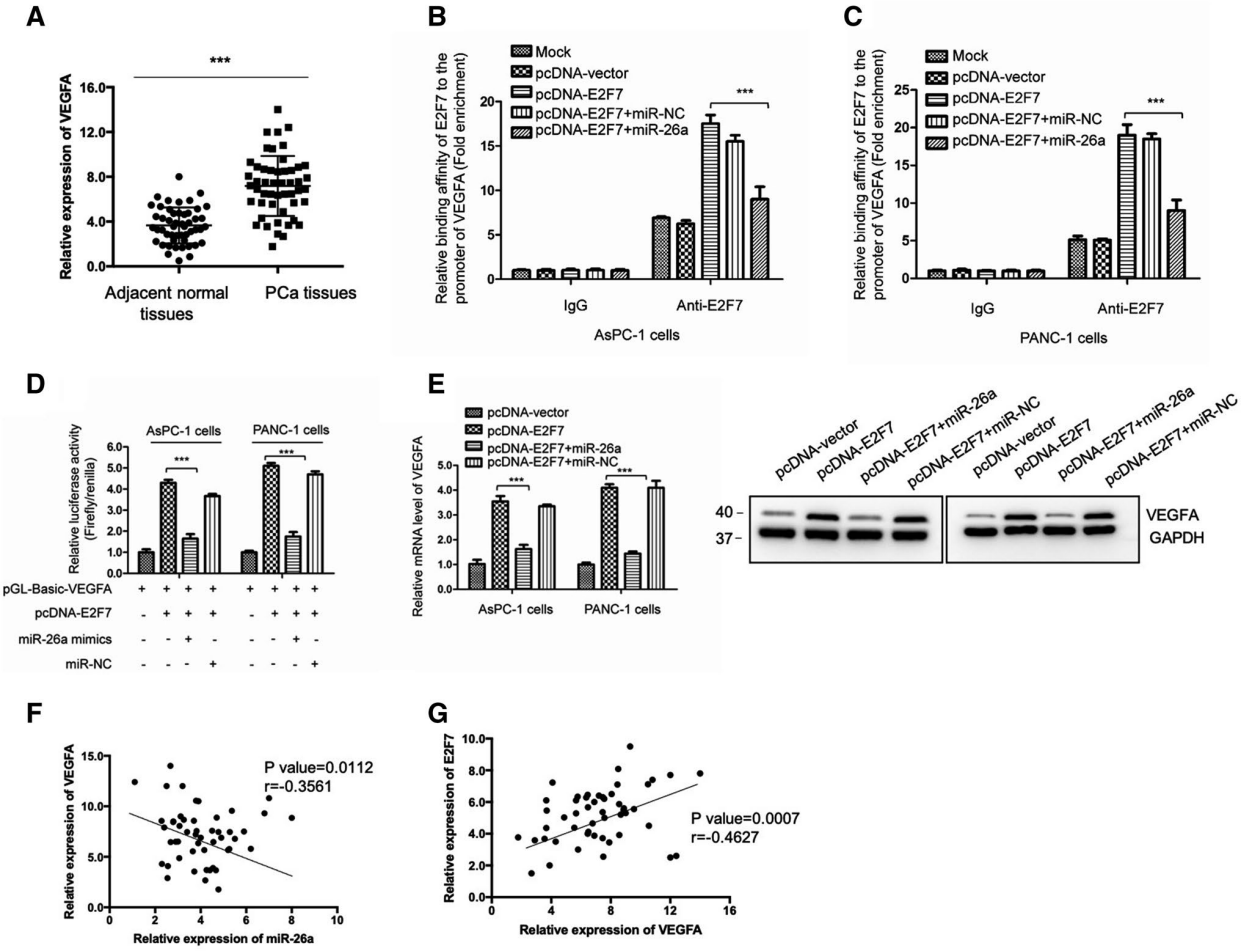
miR-26a negatively regulated E2F7-mediated transcriptional activation of VEGFA. **A** mRNA levels of VEGFA were overexpressed in PCa tissues. **B**, **C** The binding between E2F7 with the VEGFA promoter under different conditions was detected by CHIP. **D** The binding of E2F7 with the VEGFA promoter region was examined by luciferase reporter analysis. **E** The mRNA levels of VEGFA under the indicated conditions were compared via RT-qPCR. **F**, **G** The correlation between the expression of VEGFA with miR-26a or E2F7 in PCa tissues. ***P < 0.001

As a transcription factor, E2F7 can bind the promoters of target genes and regulate cancer progression. A recent study showed that E2F7 transcriptionally activated the expression of VEGFA, an important factor in angiogenesis [39]. RT-qPCR analysis showed a significantly increased expression of VEGFA in PCa tissues compared with that of the non-cancerous cohorts (Fig. 5A). To further understand the possible mechanism by which E2F7 is involved in PC, we performed a CHIP assay and found that E2F7 bound the promoter region of VEGFA in PC cells (Fig. 5B and C). However, the binding of E2F7 to the promoter region of VEGFA was reduced with miR-26a overexpression (Fig. 5B and C). We constructed a luciferase reporter vector by inserting the VEGFA promoter sequence into the backbone of the pGL3-Bacic vector and transfected this into the cells to provide more evidence for the binding between E2F7 and VEGFA. As indicated in Fig. 5D, overexpressing E2F7 dramatically increased the luciferase activity of VEGFA; however, co-transfection of miR-26a attenuated the effects of E2F7.

To explore the consequence of transcriptional inactivation of VEGFA by miR-26a, we collected cells that were transfected with miR-26a or E2F7 and compared the mRNA abundance of VEGFA. The results showed that overexpression of E2F7 increased the mRNA level of VEGFA, an effect that was abolished with co-transfection of miR-26a (Fig. 5E). This data was also supported by the western blot results (Fig. 5E, right panel). E2F7 is a transcriptional activator of VEGFA in PCa that could be negatively regulated by miR-26a. This conclusion was further supported by the findings that VEGFA expression was negatively correlated with that of miR-26a, while positively correlated with E2F7 in PCa (Fig. 5F and G).

## Discussion

PCa is a highly lethal malignancy with an extremely low five-year survival rate [40–43]. Understanding the molecular mechanisms that trigger the progression of PCa is critical to designing new biomarkers and therapeutic options. Notably, exploring the association between miRNAs and PCa tumorigenesis has been a growing cancer research field [44–48]. Several miRNAs are dysregulated in PCa, a poor prognosis factor for patients. miR-26a inhibits the proliferation, invasion, and metastasis of papillary thyroid carcinoma [49]. miR-26a suppresses the tumor metastasis of HCC by regulating the EMT process [50]. Deng et al. reported that miR-26a acted as a suppressor in pancreatic cancer via targeting cyclin E2 [34]. Decreased miR-26a expression was correlated with the aggressiveness of pancreatic cancer [34]. Additionally, locked nucleic acid (LNA)-in situ hybridization (ISH) analysis showed that miR-26a was present in the cytoplasm of pancreatic ductal epithelial cells [34]. This data is also consistent with our knowledge that miRNA is processed from the precursor pri-miRNA in the nucleus, where pri-miRNA is cleaved into pre-miRNA and then transport into the cytoplasm to mature into miRNA. Consistent with this study, we found the reduced expression of miR-26a in PCa. Ectopically expressed miR-26a suppressed growth and stemness features and activated the apoptosis of PCa cells. Consistent with previous reports, this study demonstrated the tumor-suppressive function of miR-26a in PCa.

E2F7 belongs to the E2F transcription factor family. As an atypical E2F transcription factor, E2F7 functions as a key regulator of cell cycle progression, and its inactivation leads to spontaneous cancer formation [51]. The frequent deregulation of E2F7 in human cancers has been established. A low level of E2F7 predicted poor survival of patients with glioma and might constitute a potential therapeutic target for glioma [52]. Interestingly, it was also reported that E2F7 was a tumor-promoting factor in breast cancer, inducing cancer cell proliferation, invasion, and metastasis [53]. Therefore, the opposite function of E2F7 in different cancers relies on individual cancer types.

Our results demonstrated the overexpression of E2F7 in PCa tissues, a reversible trend compared with miR-26a expression. Consistent with this observation, our finding also indicated the inhibitory effect of miR-26a on E2F7 via binding of the 3’-UTR of E2F7, suggesting E2F7 as a target of miR-26a in PCa. The reintroduction of E2F7 significantly attenuated the inhibitory effects of miR-26a on the proliferation of PCa cells. miR-26a overexpression still had a suppressive effect in E2F7 knockout cells, suggesting E2F7 partially mediated the function of miR-26 in PCa. In addition to E2F7, other potential targets of miR-26a were also predicted and they may also partially contribute to the function of miR-26a in pancreatic cancer, which deserves further investigation. E2F7 directly binds and transcriptionally activates VEGFA to control the angiogenesis [54]. VEGF family members, especially VEGFA, have been found to play important role in pancreatic cancer [54]. As the most specific and major angiogenic factor, VEGFA overexpression was suggested as a diagnostic and prognostic factor for pancreatic cancer [54]. A further mechanism study revealed that E2F7 bound the promoter of VEGFA and transcriptionally activated its expression, a process that can be inhibited by miR-26a. This result provided the possible mechanism by which E2F7 is involved in the progression of PC. To further demonstrate the essential of VEGFA in the functional effects of miR-26a-E2F7, in vivo xenograft mouse model was established by injecting VEGFA-/- PCa cells. The primary data indicated that compare with wild-type cells, overexpressed miR-26a significantly lost it tumor suppressive effects in the absence of VEGFA (data not shown). Further experiments are still ongoing to clarify the essential role of VEGFA in the functional effects of miR-26a/E2F7. It is well known that E2F7 has a variety of targets which can be regulated under different conditions [55–58]. In addition to VEGFA, Harmonizome website (https://maayanlab.cloud/Harmonizome/) was used to search more potential targets of E2F7. 46 target genes of E2F7 were found in low or high-throughput transcriptional factor functional studies from the CHEA Transcription Factor Targets database. The involvement of these targets of E2F7, such as BRCA1, CDC25A, CDC6 in pancreatic cancer deserves further investigation. Given the aberrant expression of miR-26a and E2F7 in PCa, it would be interesting to evaluate their clinical significance in the diagnosis or prognosis of PCa patients. Importantly, there are different targets of miR-26a reported so far. The function of other targets and how they work together with E2F7 in the development of PCa requires further investigation.

## Conclusions

Our study uncovered the down-regulation of miR-26a in PCa and that miR-26a inhibited the malignant behaviors of PCa cells by targeting E2F7. These findings provide a promising strategy for overcoming PCa by blocking miR-26a.
